# Calcium‐ and Tyrosine Kinase‐Dependent Suppression of Macroscopic Homomeric Kv1.2 and Heteromeric Kv1.1/1.2 current by Amyloid Beta (1‐42) Peptide: Implications for Alzheimer’s Disease

**DOI:** 10.1002/alz.087563

**Published:** 2025-01-03

**Authors:** Dina Jamshidi, Logen Yaklin, Sebastian Burch, Kristi DeBoeuf, Joseph Farley

**Affiliations:** ^1^ Indiana University, Bloomington, IN USA

## Abstract

**Background:**

The roles of Aβ in the pathogenesis of Alzheimer ’s disease (AD) include disruption of synaptic communication/function and synaptic plasticity mechanisms thought to underlie learning and memory. Exactly how these abnormal processes arise is incompletely understood, but evidence suggests that dysregulation of intracellular Ca^2+^ levels is involved in alterations of neuronal excitability, synaptic remodeling, and neurodegeneration in AD. Our lab has focused on the potential involvement of voltage‐gated potassium channels (VGKCs) in these processes, particularly Kv1.x family members. VGKCs contribute to resting membrane potential, regulate Ca^2+^ influx, and their inhibition may lead to and synapto‐ and neuro‐toxicity through hyperexcitability and excess glutamate release. Kv1.x family members are expressed as homotetramers, or as select heterotetramers (e.x Kv1.1/1.2) contributing to the diversity of electrical signaling. Previously, we have observed rapid and robust suppression (50% in 30 minutes) of macroscopic current by Aβ(1‐42) in homomeric Kv1.1 expressing *Xenopus laevis* oocytes. We sought to understand 1) if suppression by Aβ(1‐42) is exclusive to Kv1.1, or is able to affect other Kv1.x members such as homomeric Kv1.2 and heteromeric Kv1.1/1.2 channels 2) The extent to which the suppression is Ca^2+^ dependent, and 3) whether the suppression is additionally dependent on endogenous tyrosine kinase activity.

**Method:**

Stage V/VI *Xenopus laevis* oocytes were injected with Kv1.2 cRNA or both Kv1.1 and 1.2 cRNA for homomeric and heteromeric expression respectively. The effects of bath application of 1 μM of Aβ(1‐42) were evaluated using two‐electrode voltage clamp electrophysiology (TEVC), and Ca^2+^ dependency was evaluated using BAPTA‐AM (a Ca^2+^ chelator) and Cyclosporin A (CsA; a PP2B inhibitor). Oocytes were additionally incubated in genistein, a non‐specific tyrosine kinase inhibitor.

**Result:**

For both homo‐and heteromeric channel expressing oocytes, incubation in BAPTA‐AM significantly reduced suppression by ∼45%, while incubation with CsA blocked any suppressive effects of Aβ(1‐42). Oocytes incubated in genistein significantly blocked Aβ‐suppression at a level greater than CsA and BAPTA‐AM (>98%).

**Conclusion:**

Reducing intracellular Ca^2+^ and inhibiting Ca^2+^ activated signaling molecules such as PP2B and tyrosine kinases reduces suppression of macroscopic Kv1.x current by Aβ(1‐42).

